# Epidemiology of Locomotive Organ Disorders and Symptoms: An Estimation Using the Population-Based Cohorts in Japan

**DOI:** 10.1007/s12018-016-9211-7

**Published:** 2016-06-07

**Authors:** Noriko Yoshimura, Kozo Nakamura

**Affiliations:** Department of Joint Disease Research, 22nd Century Medical and Research Center, The University of Tokyo, Hongo 7-3-1, Bunkyo-ku, Tokyo, 113-8655 Japan; National Rehabilitation Center for Persons with Disabilities, Saitama, 359-0042 Japan

**Keywords:** ROAD study, Osteoarthritis, Osteoporosis, Knee pain, Lumbar pain, Locomotive syndrome

## Abstract

Although locomotive organ diseases such as osteoporotic fractures and osteoarthritis are major reasons for disability and require support, little information is available regarding the epidemiology of musculoskeletal dysfunction and its symptoms including knee pain and lumbar pain in Japan. The research on osteoarthritis/osteoporosis against disability (ROAD) study is a prospective cohort study that aims at elucidating the environmental and genetic background for locomotive organ diseases, and has been ongoing since 2005. In this review, epidemiological indices such as prevalence of locomotive organ diseases including knee osteoarthritis, lumbar spondylosis, and osteoporosis were clarified using baseline survey results of the ROAD study. The number of subjects with such diseases was estimated. In addition, 3-year follow-up data from the ROAD study revealed the effect of osteoarthritis on the occurrence of osteoporosis, and vice versa. The prevalences of osteoarthritis and osteoporosis were shown to be high. Also, the large estimates of patients with these conditions suggest that urgent strategies are needed for addressing locomotive organ diseases that cause disability in the elderly. We also clarified the prevalence of knee pain, lumbar pain, and their co-existence using the survey results of the longitudinal cohorts of motor system organ study. We found that both knee pain and lumbar pain were prevalent in 12.2 % of the total population and the presence of knee pain affected lumbar pain, and vice versa.

## Introduction

Locomotive organ disorders involving musculoskeletal dysfunction, including osteoarthritis (OA) and osteoporosis (OP), are major public health problems among the elderly. These disorders can affect mobile function, activities of daily living and quality of life. According to the most recent National Livelihood Survey by the Ministry of Health, Labour, and Welfare in Japan, osteoporotic fracture and falls are ranked fourth and OA is ranked fifth among conditions that cause disability and subsequently require support with regard to the activities of daily living [1].

Given the increasing proportion of elderly individuals in the Japanese population, a comprehensive and evidence-based prevention strategy for locomotive organ disorders is urgently required. The Japanese Orthopaedic Association proposed that the term ‘locomotive syndrome’ be adopted to designate a condition requiring nursing care, or the risk of developing such a condition, following a decline in mobility resulting from one or more disorders of the locomotive system, which consists of bones, joints, muscles, and nerves [2]. The weakness of locomotive components causes difficulty in mobility, which is defined as the ability to stand, walk, run, climb stairs, and perform other physical functions essential to daily life.

Only a few prospective, longitudinal studies have been conducted in this area. Therefore, in Japan, little information is available regarding the epidemiology of locomotive organ disorders, including knee OA (KOA), lumbar spondylosis (LS), osteoporosis (OP) at the lumbar spine L2-4 and femoral neck, and their symptoms such as knee pain and lumbar pain.

The research on osteoarthritis/osteoporosis against disability (ROAD) study, which started in 2005–2007, is a prospective cohort study that aims at elucidating the environmental and genetic background for bone and joint diseases including OA and OP. It is designed to examine the extent to which risk factors for these diseases are related to clinical features, laboratory and radiographic findings, bone mass and bone geometry, lifestyle, nutritional factors, anthropometric and neuromuscular measures, and fall propensity as well as to determine how these diseases affect activities of daily living and quality of life in Japanese men and women [3]. The 3-year follow-up (the 2nd survey) was performed on residents of the same communities in 2008–2010, the 7-year follow-up (the 3rd survey) was carried out in 2012–2013, and the 10-year follow-up is now in progress.

In this review, epidemiological indices such as the prevalence of locomotive organ diseases such as KOA, LS, and OP were clarified using the baseline study results and 3-year follow-up results of the ROAD study. For knee and lumbar pain, we used the results of the longitudinal cohorts of motor system organ (LOCOMO) study, details of which are described later.

## Prevalence of Locomotive Organ Disorders

In this section, we cite the results of above-mentioned ROAD study. Recruitment methods for this study have been described in detail elsewhere [3, 4]. We have a baseline database that includes clinical and genetic information of 3040 inhabitants (1061 men and 1979 women) aged 23–95 years who were recruited from the resident registrations listings of three communities. All participants provided written informed consent, and the study was conducted with approval from the ethics committees of the participating institutions.

### Prevalence of KOA and LS

Plain radiographs with anteroposterior and lateral views of the lumbar spine and an anteroposterior view of the bilateral knees and hips with weight-bearing and foot map positioning were obtained. The severity of radiographic OA was determined according to the Kellgren–Lawrence (KL) scale [5]. Radiographs of the knees, hips, and vertebrae were examined by a single, experienced orthopaedic surgeon (knees and vertebra, S.M.; hips, T.I.) who was unaware of the participants’ clinical status. If at least one joint was graded as KL2 or higher, the participant was diagnosed with radiographic OA.

**Fig. 1:**
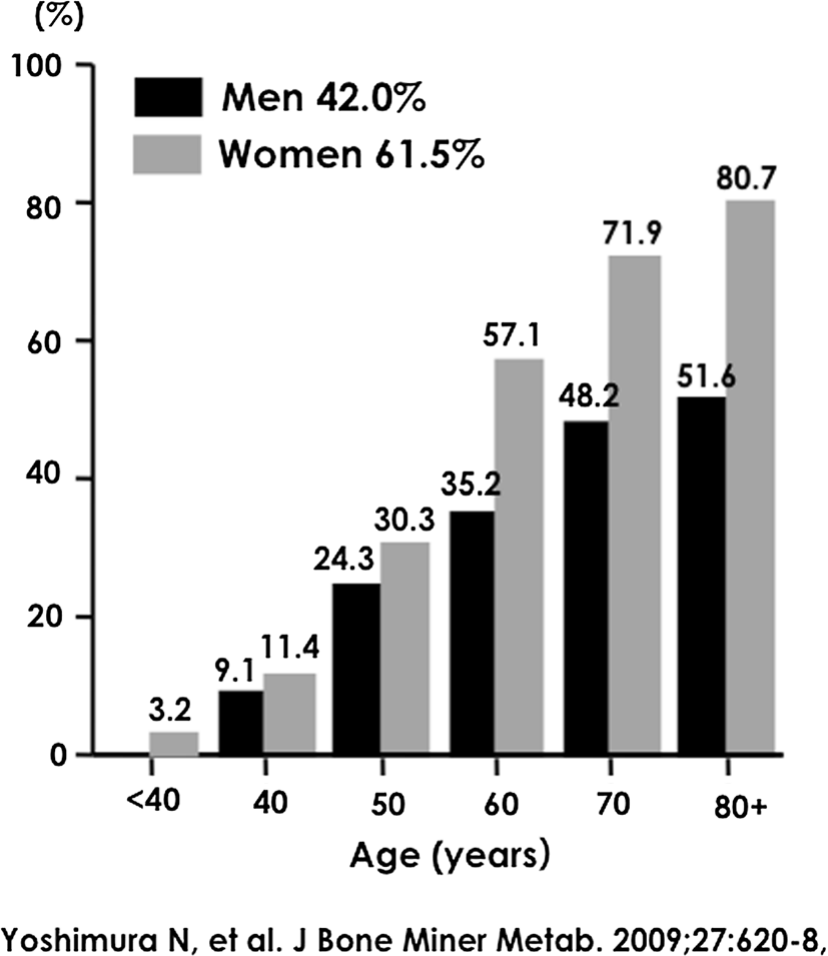
Prevalence of knee osteoarthritis with a Kellgren–Lawrence grade ≥2

Figure 1 shows the age–sex distribution for prevalence of radiographic KOA as determined by a KL grade ≥2. In the overall population, prevalence of radiographic KOA was 54.6 % (42.0 % in men and 61.5 % in women). Radiographic KOA tended to be higher with age in both genders. Moreover, in the overall population, the prevalence was significantly higher in women than in men (*p* < 0.001).

**Fig. 2:**
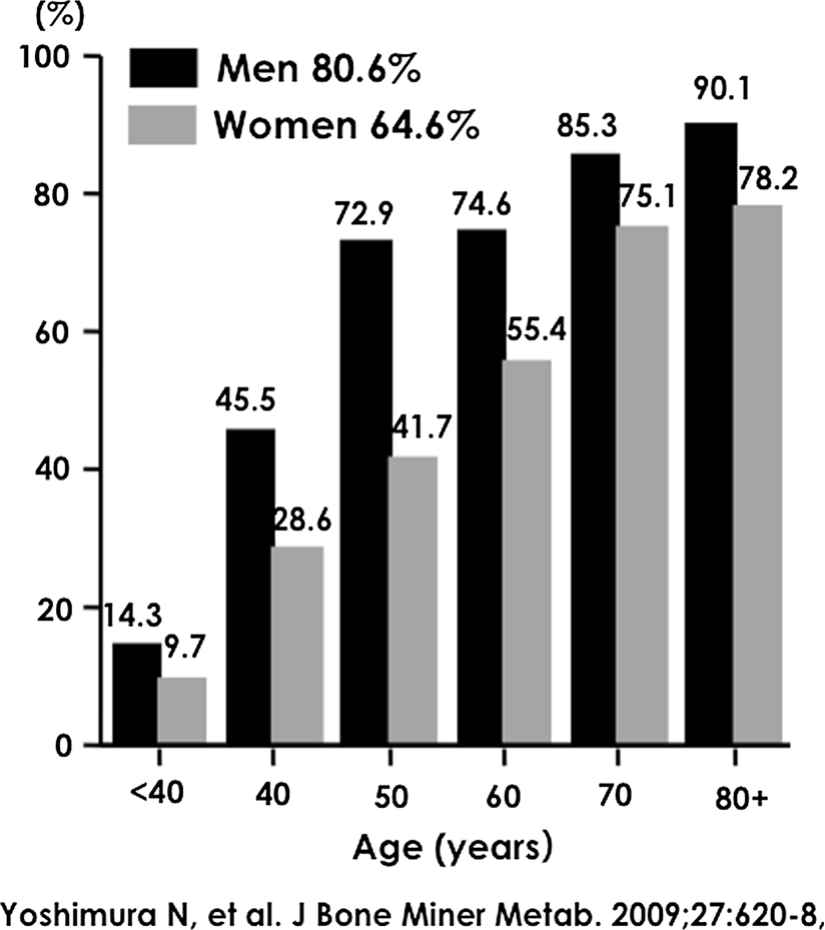
Prevalence of lumbar spondylosis with a Kellgren–Lawrence grade ≥2

Similarly, Fig. 2 shows the prevalence of radiographic LS as determined by a KL grade ≥2. The prevalence of radiographic LS was 70.2 % (80.6 % in men and 64.6 % in women), that is, the prevalence tended to be higher with age in both genders. Unlike radiographic KOA, the prevalence was significantly higher in men than in women (*p* < 0.001).

The present study clarified the age–sex distribution of the prevalence of KOA and LS as diagnosed radiographically in Japanese populations. If the ROAD study results were applicable to the total age–sex distribution derived from the Japanese census in 2005 [7], we can assume that 25,300,000 (8,600,000 men and 16,700,000 women) and 37,900,000 (18,900,000 men and 19,000,000 women) people aged 40 years and older would be affected by radiographic KOA and LS, respectively. This estimation includes asymptomatic OA. Since one-quarter of the men and one-third of the women with radiographic OA were reported to have pain, a feature of symptomatic OA [8, 9], approximately 7,800,000 people (2,200,000 men and 5,600,000 women) aged 40 and older are expected to be affected by symptomatic KOA. Similarly, 11,000,000 people (4,700,000 men and 6,300,000 women) are estimated to be affected by symptomatic LS.

### Prevalence of OP

For all 1,690 of the 3040 ROAD baseline survey participants from mountainous and coastal areas, bone mineral density (BMD) was measured at the lumbar spine L2-4 and the proximal femur using dual-energy X-ray absorptiometry (DXA; Hologic Discovery, Hologic, Waltham, MA, USA). The same physician (N.Y.) examined all participants to prevent observer variability. OP was defined as a BMD of less than 70 % of peak bone mass according to the criteria of the Japanese Society for Bone and Mineral Research [6]. A BMD of <0.708 g/cm^2^ at the lumbar spine in both men and women, a BMD of <0.604 g/cm^2^ at the femoral neck in the case of men, and <0.551 g/cm^2^ in the case of women were all defined as OP.

**Fig. 3:**
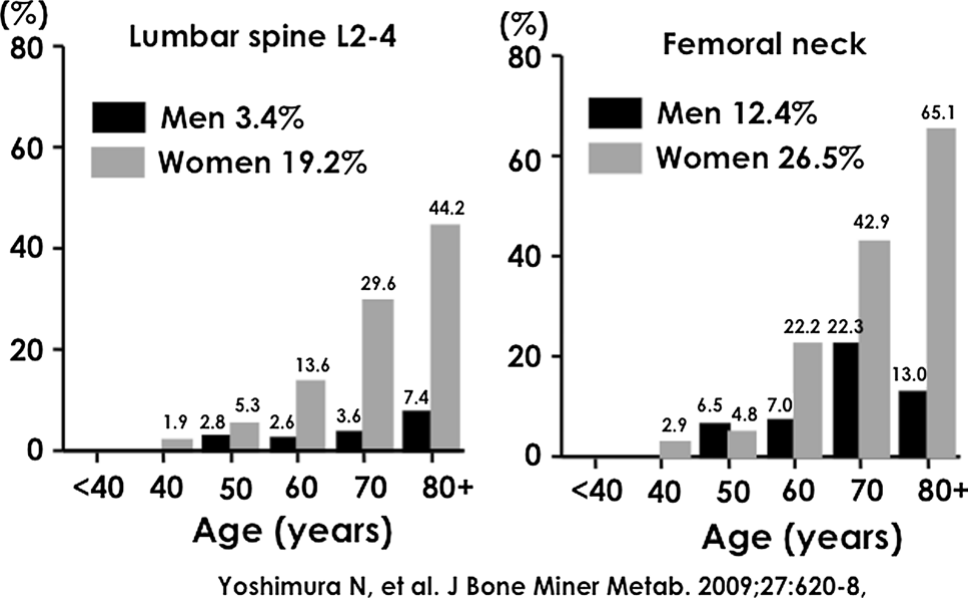
Prevalence of osteoporosis according to the criteria of the Japanese Society for Bone and Mineral Research

Figure 3 reveals the prevalence of OP at the lumbar spine and femoral neck among residents of mountainous and coastal regions in the ROAD study. The prevalence of OP at the lumbar spine and femoral neck in women was sixfold and twofold significantly higher, respectively, than in men (*p* < 0.001). Considering the total age–sex distribution according to the Japanese census in 2005 [7], we can assume that 6,400,000 people (800,000 men and 5,600,000 women) aged 40 years and older had OP at the lumbar spine L2-4, while 10,700,000 (2,600,000 men and 8,100,000 women) of the same cohort had OP at the femoral neck.

The prevalence of the presence of OP either at the lumbar spine L2-4 or femoral neck in participants aged <40, 40–49, 50–59, 60–69, 70–79, and ≥80 years was 0.0, 2.7, 8.2, 20.8, 39.8, and 54.4 %, respectively (0.0, 0.0, 9.4, 7.6, 23.6, and 16.7 %, respectively, in men and 0.0, 3.8, 7.7, 27.2, 50.9, and 74.0 %, respectively, in women). Further, based on the total age and sex distributions according to the Japanese census in 2005 [7], 12,800,000 people (3,000,000 men and 9,800,000 women) aged 40 years and older were affected by OP at either the lumbar spine L2-4 or femoral neck.

### Co-existence of KOA, LS, and OP

**Fig. 4:**
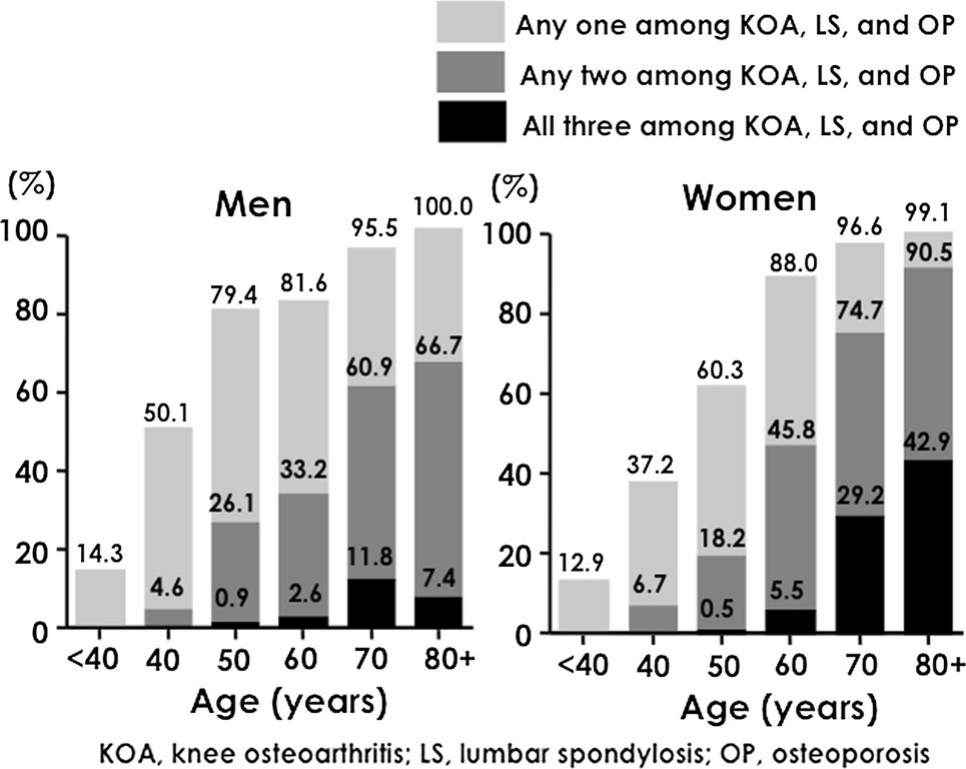
Prevalence classified by the number of knee osteoarthritis, lumbar spondylosis, and osteoporosis cases

Figure 4 shows the prevalence of either OA (KOA or LS) or OP classified by sex and age. If the ROAD study results were applicable to the total age–sex distribution from the Japanese census in 2005 [7], we can assume that 47,000,000 people (21,000,000 men and 26,000,000 women) aged 40 years and older were affected by KOA, LS, or OP. Considering this large number of patients, strategies for addressing these locomotive organ disorders that cause disability in the elderly must be urgently drafted and applied.

## The Effect of Osteoarthritis on the Occurrence of Osteoporosis and Vice Versa

**Fig. 5:**
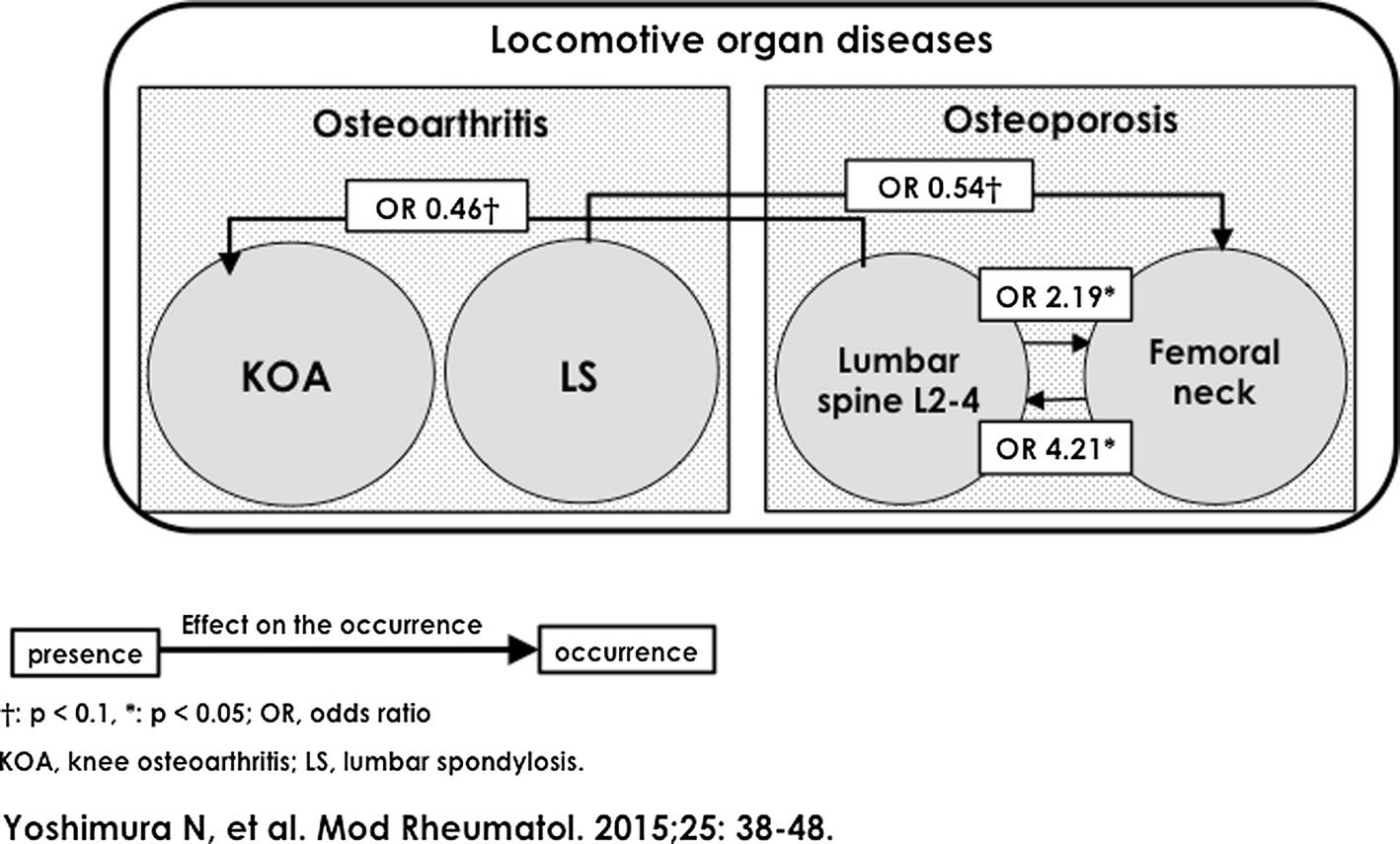
Effect of the presence of locomotive organ diseases on the occurrence of other locomotive organ diseases

Of the 1690 participants in the baseline survey that was performed in mountainous and coastal regions, 1384 subjects (81.9 %; 466 men and 918 women) completed all the examinations, both at baseline and 3-year follow-up. Using the corresponding information, mutual causal associations between the occurrence of locomotive organ diseases such as KOA, LS, and OP at the lumbar spine and femoral neck were explored [10]. Figure 5 reveals the mutual associations between OA and OP [10]. The risk of the occurrence of OP at L2-4 was increased by the presence of OP at the femoral neck (*p* < 0.01), and the risk of the occurrence of OP at the femoral neck was increased by the presence of OP at L2-4 (*p* < 0.05). The presence of OP at lumbar L2-4 tended to decrease the risk of the occurrence of KOA, and the presence of LS tended to decrease the risk of the occurrence of OP at the femoral neck, although both of these associations were not statistically significant.

## Prevalence of Knee Pain and Lumbar Pain and Their Co-existence

The prevalence of knee pain and lumbar pain was determined from the survey results of the longitudinal cohorts of motor system organ (LOCOMO) study [11]. The LOCOMO study was initiated in 2008 through a grant from the Ministry of Health, Labour, and Welfare in Japan to integrate information from several cohorts established for the prevention of locomotive organ diseases. The LOCOMO study integrated information of 12,019 participants (3959 men and 8060 women) in cohorts comprising nine communities located in Tokyo (two regions), Wakayama (two regions), Hiroshima, Niigata, Mie, Akita, and Gunma prefectures. The three communities from the ROAD study were also involved in the LOCOMO study. The LOCOMO study participants were questioned about pain in both knees through the following questions: ‘Have you experienced right knee pain on most days (and continuously on at least 1 day) in the past month, in addition to the current pain?’ and ‘Have you experienced left knee pain on most days (and continuously on at least 1 day) in the past month, in addition to the current pain?’. Subjects who answered ‘yes’ were considered to have knee pain. The presence of lumbar pain was determined by asking the following question: ‘Have you experienced lumbar pain on most days (and continuously on at least 1 day) in the past month, in addition to the current pain?’. Subjects who answered ‘yes’ were considered to have lumbar pain.

**Fig. 6:**
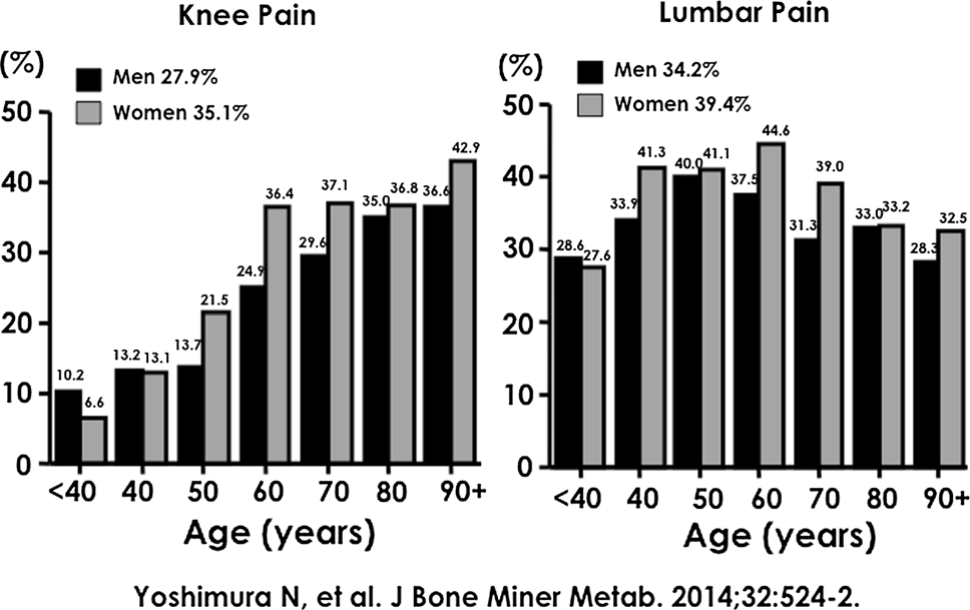
Prevalence of knee pain and lumbar pain

Figure 6 shows the prevalence of knee and lumbar pain. The prevalence rate of knee pain was 32.7 % (men, 27.9 %; women, 35.1 %) and that of lumbar pain was 37.7 % (men, 34.2 %; women, 39.4 %) [9]. On the basis of the total age and sex distributions derived from the Japanese census in 2010 [10], our results estimate that 18,000,000 (7,100,000 men and 10,900,000 women) and 27,700,000 (12,100,000 men and 15,600,000 women) people aged ≥40 years would be affected by knee pain and lumbar pain, respectively.

Among the 9046 individuals who were surveyed for both regarding knee pain and lumbar pain at the baseline examination in each cohort, we noted that the prevalence of both knee pain and lumbar pain was 12.2 % (men, 10.9 %; women, 12.8 %) [11]. The prevalence of the co-existence of knee and lumbar pain in participants aged <40, 40–49, 50–59, 60–69, 70–79, and ≥80 years was 4.0, 4.8, 7.4, 13.0, 13.3, and 11.7 %, respectively (6.1, 5.3, 6.0, 10.0, 11.5, and 13.2 %, respectively, in men and 2.6, 4.6, 8.1, 14.8, 14.2, and 11.0 %, respectively, in women). On the basis of the total age and sex distributions derived from the Japanese census in 2010 [12], 6,800,000 people (2,800,000 men and 4,000,000 women) aged ≥40 years would be affected by both knee pain and lumbar pain.

## Conclusion

As shown, there was little information regarding the epidemiology of locomotive organ disorders such as OA and OP, and their symptoms such as knee and lumbar pain in Japan. The ROAD study is the first large observational study conducted in the Japanese population that was designed to supply essential information mainly regarding OA and OP. Among the large-scale population-based epidemiological studies aimed at preventing OA, the ROAD study, which includes 3040 participants, ranks first compared to the Framingham study with 1805 participants [13] and the Chingford study with 1353 participants [14].

In the present review, which uses the latest reports of population-based epidemiological studies such as the ROAD and LOCOMO studies, the prevalence of locomotive organ diseases and symptoms was clarified. Determining the frequency of a disorder is the first step to preventing it. Hence, prevalence may be useful for determining a reduced target value in future interventional or observational studies with the aim of preventing disability among inhabitants in general. In addition, the prevalence of the co-existence of these diseases was very high. Therefore, there is an urgent need to draft and apply strategies for addressing the locomotive organ diseases that cause disability in the elderly.

The LOCOMO study showed that the prevalence of knee pain was 32.7 % and that of lumbar pain was 37.7 %. Both knee pain and lumbar pain were prevalent in 12.2 % of the total population. In addition, the presence of knee pain affected that of lumbar pain and vice versa.

In conclusion, we clarified the prevalence of KOA, LS, and OP and estimated the number of people affected in Japan using the baseline data of the ROAD study. The study will provide the information required to develop clinical algorithms for the early identification of potential high-risk populations and policies for the detection and prevention of OA, OP, or osteoporotic fractures. Furthermore, establishment of the cohort will also facilitate the expansion of other studies in related areas of investigation. The knowledge gained from both the ROAD study and the LOCOMO study will have major implications for the understanding and management of several other common problems of ageing.
